# Chain mediation effects of depressive symptoms and nutritional risk between social support and oral frailty in patients with coronary heart disease

**DOI:** 10.3389/fpubh.2026.1880497

**Published:** 2026-07-14

**Authors:** Yufeng Wang, Qingqiu Gan, Juan Zhang, Meiyi Tao

**Affiliations:** 1 School of Nursing, Hunan Normal University, Changsha, Hunan, China; 2 Department of Nursing, Hunan Provincial People’s Hospital (The First Affiliated Hospital of Hunan Normal University), Changsha, Hunan, China

**Keywords:** coronary heart disease, depression, nutrition, oral frailty, social support

## Abstract

**Introduction:**

Oral frailty among coronary heart disease (CHD) patients is prevalent and harmful. CHD patients have encountered serious physical and psychological health issues. Social support is a crucial protective factor in chronic disease management. There is a paucity of evidence regarding the specific association among CHD patients. Depressive symptoms and nutritional risk have been demonstrated to be associated with oral frailty, but the mediating role between them under the influence of social support remains to be further explored. This study aimed to investigate the chain mediating effect of depressive symptoms and nutritional risk between social support and oral frailty among CHD patients.

**Methods:**

This is a cross-sectional study involving 500 hospitalized CHD patients, conducted from September 2025 to January 2026. The assessment tools used included a demographic characteristics, the Social Support Rating Scale (SSRS), the PHQ-9 Depression Screening Scale (PHQ-9), the Nutritional Risk Screening 2002(NRS 2002), and the Oral Frailty Index-8 (OFI-8). Pearson correlation analysis was used to examine relationships among variables, and the Bootstrap method (with 5,000 repeated samples) was employed to test for mediating effects.

**Results:**

The prevalence of oral frailty among CHD patients was 57.60%. The mean scores for social support, depressive symptoms, nutritional risk, and oral frailty were (37.67 ± 8.15), (13.41 ± 3.47), (1.12 ± 1.13), and (4.80 ± 2.86), respectively. Correlation analysis revealed that oral frailty was negatively correlated with social support and positively correlated with depressive symptoms and nutritional risk. Mediation analysis indicated that social support not only directly influences oral frailty but also indirectly influenced it through depressive symptoms, nutritional risk, and the chain path between depressive symptom and nutritional risk; the chain mediation effect accounted for 15.57% of the total effect. After adjusting for covariate, the mediation effects remained statistically significant.

**Discussion:**

The study showed that social support can predict oral frailty through depressive symptoms and nutritional risk. It is recommended that healthcare professionals provide comprehensive interventions to enhance social support for CHD patients and actively manage their depressive symptoms and nutritional risks to prevent the progression of oral frailty.

## 1 Introduction

Coronary heart disease (CHD) imposes a widespread, adverse health burden on affected patients. Research indicates that CHD incidence is rising year by year, making it the leading cause of death from cardiovascular diseases (1, 2). Oral frailty (OF) refers to an age-related multidimensional decline in oral function, including tooth loss, impaired masticatory and swallowing function, and altered oral health behaviors, affecting both physiological and psychological domains (3). OF is closely related to systemic chronic inflammation, malnutrition, and cognitive decline, and may exacerbate the cardiovascular deterioration through pathways such as sustained low grade inflammation, oral microbiota translocation, and compromised nutritional intake (4). Increasing evidence indicates an association between oral health and adverse cardiovascular events (5, 6). Furthermore, polypharmacy is highly prevalent among CHD patients. The volume and flow rate of saliva secretion decrease as the number of consumed medications increases (7–9). This particularity makes CHD patients more susceptible to oral problems, thereby resulting in oral frailty.

For CHD patients, the physical, psychological, and social burden is substantial. The Gobbens’ integrative theory of frailty posits that frailty is a dynamic integration of physical, psychological, and social dimensions, with each dimension interacting with one another and correlating with adverse outcomes (10, 11). Research indicates that higher level of social support can delay the progression of oral frailty. Individuals with better social support have more dietary choices and better muscle maintenance capabilities, suggesting that social support may play a protective role in oral frailty by improving nutritional status (12, 13). In addition, previous studies have indicated that various psychological factors mediate health outcomes, particularly depressive symptoms. Insufficient social support can directly increase the risk of depression. Depressive symptoms can accelerate the destruction of periodontal tissues through mechanisms such as immunosuppression and inflammatory dysregulation (14–16). Additionally, patients with depression frequently exhibit self-neglect, and their oral self-efficacy is reduced (17); thus, depressive symptoms are hypothesized to act as a mediator between social support and oral frailty. Another influencing factor of oral frailty is nutritional risk. Malnutrition decrease the repair capacity of the oral mucosa and alter saliva composition, accelerating the progression of oral frailty (18). Notably, depressive symptoms can further worsen nutritional status. The taste disorders that accompany depressive symptoms reduce nutrition intake (19). This subsequently leads to the atrophy of oropharyngeal muscles and a decline in chewing efficiency, ultimately manifesting as further oral frailty (20, 21). Taken together, social support, depressive symptoms, and nutritional risk represent interconnected modifiable determinants, with a plausible chain pathway from social support to depressive symptoms, then to nutritional risk, and finally to oral frailty.

Despite growing recognition of oral frailty as associate factor to physical frailty and adverse cardiovascular outcomes, existing literature has predominantly focused on community-dwelling or general geriatric populations. Hospitalized CHD patients, however, experience distinct changes in their daily environment and social interaction patterns, and face concentrated risks of depression and nutritional deficiency during hospitalization. This acute clinical setting represents a critical window for targeted intervention, yet evidence regarding the association between social support and oral frailty in this specific population remains scarce.

To date, few studies have examined the association between social support and oral frailty in CHD patients, and none have systematically investigated the mediating pathways involving depressive symptoms and nutritional risk, particularly the chain mediating effect. Accordingly, the present study aimed to characterize the relationship between social support and oral frailty in CHD patients, and to test the hypothesis that social support exerts both a direct protective effect and an indirect effect via the mediation of depressive symptoms and nutritional risk. This work will generate actionable evidence to inform the development of multifaceted clinical interventions aimed at mitigating oral frailty risk and improving long term health outcomes in the CHD patients. The hypotheses are as follows:

*H1*: Social support and oral frailty among patients with CHD have a significant negative correlation.

*H2*: Depressive symptoms mediate the relationship between social support and oral frailty.

*H3*: Nutritional risk mediates the relationship between social support and oral frailty.

*H4*: Depressive symptoms and nutritional risk play a chain mediating effect in the relationship between social support and oral frailty.

## 2 Methods

### 2.1 Design and participants of the study

This cross-sectional study aimed to explore the mediation effects between social support and oral frailty. A convenience sampling method was employed to recruit hospitalized CHD patients from the Department of Cardiology at a tertiary Grade A hospital in Changsha, Hunan, China. Data collection took place between September 2025 and January 2026, and standardized data collection procedures were strictly followed throughout the process to ensure the accuracy and completeness of the information gathered. The inclusion criteria were: diagnosed with coronary heart disease; conscious, possessing basic language communication and cognitive judgment abilities; providing informed consent and volunteering to participate in this study. The exclusion criteria were: patients currently participating in other clinical studies; with severe mental disorders or language communication barriers; diagnosed with other severe organic lesions; with concurrent heart diseases of other types.

All participants freely and voluntarily joined the study. Prior to the survey, uniformly trained data collectors explained the research objectives, content, and confidentiality protocols to ensure participants were fully informed. Questionnaires were checked on the spot for completeness upon submission. Data should be entered only after being cross-checked by two people. Based on Kendall’s multivariate sample size estimation, the final analysis sample for this study included 500 patients. The study procedures were conducted after obtaining formal consent from the hospital.

### 2.2 Instruments

A survey was administered via paper questionnaires to measure various variables, including:

#### 2.2.1 Demographic characteristics

A general information questionnaire was designed based on the research objectives, and the content included: age, gender, education, monthly household income per person, residence, number of missing teeth, polypharmacy, course of coronary heart disease.

#### 2.2.2 Social support rating scale (SSRS)

SSRS was developed by Xiao Shuiyuan and consists of 10 items across 3 dimensions: subjective support (4 items),objective support (3 items), and support utilization (3 items) (22). Items 1–4 and 8–10 are single choice; selections 1–4 are scored 1–4 points, respectively. Item 5 is calculated as the sum of its four sub-items, each scored from 1 (“none”) to 4 (“full support”); Items 6 and 7, a response indicating “no source” scores 0 points, while choosing “the following sources” scores 1 point for each source selected. The total score is the sum of all item scores, ranging from 12 to 66. Higher scores indicating a greater perceived adequacy of social support. The total score ≤22 indicates low level support, 23–44 indicates moderate level support, and 45–66 indicates high level support. In this study, its Cronbach’s *α* was 0.802, indicating good reliability consistent with the validated version (23).

#### 2.2.3 Patient health questionnaire-9 (PHQ-9)

The PHQ-9 was developed from the 9 symptomatic criteria of the American Diagnostic and Statistical Manual of Mental Disorders, is widely used for preliminary diagnosis and severity assessment of depression (24). It comprises 9 items, each scored on a 4-point scale from 0 to 3, where 0 indicates “not at all” and 3 indicates “almost every day.” A total score≥5 indicates depression. Scores of 5–9 indicate mild depression, 10–14 indicate moderate depression, 15–19 indicate moderately severe depression, and 20–27 indicate severe depression. In the present study, the Cronbach’s *α* of this scale was 0.804, indicating good internal consistency reliability (25).

#### 2.2.4 Nutritional risk screening 2002 (NRS 2002)

NRS 2002 was developed by Kondrup in 2002 and recommended by the Chinese Society for Parenteral and Enteral Nutrition (CSPEN) as a standardized nutritional screening tool for hospitalized patients (26). It comprises three dimensions: nutritional status (a score of 0 is assigned for no weight loss in the past 3 months, 1 for weight loss > 5% in the past 3 months, 2 for weight loss > 5% in the past 2 months, and 3 for weight loss > 5% in the past 1 month), disease severity (a score of 0 corresponds to normal status, 1 to chronic diseases with complications, 2 to moderate conditions such as major abdominal surgery, and 3 to severe conditions such as craniocerebral injury), and age (a score of 0 is given for subjects younger than 70 years and 1 for those aged 70 years and above). The first two dimensions are scored from 0 to 3 in ascending order of severity, while the age dimension is scored from 0 to 1. The total score ranging from 0 to 7 points, and a total score of ≥3 indicates nutritional risk, with higher scores reflecting a higher level of nutritional risk. The reliability of the scale in this study was 0.758, which is considered good (27).

#### 2.2.5 Oral frailty index-8 (OFI-8)

The OFI-8 was developed by the Japan Dental Association and adapted for Chinese use by Tanaka et al. (4) and Chen et al. (28). This scale comprises five dimensions: denture use, swallowing function, social participation, oral health behaviors, and masticatory ability. It consists of eight items with a total score range of 0–11 points. A score of ≥4 indicates oral frailty. The scale has good reliability and validity, with Cronbach’s *α* is 0.832 in this study (29).

### 2.3 Statistical analysis

Descriptive statistics were adopted for data summarization: categorical variables were expressed as frequencies and percentages, and continuous variables as means and standard deviations. Pearson correlation analysis was conducted. Mediation effects were examined using Model 6 of the PROCESS V4.1 plugin. In this model, social support is defined as the independent variable (*X*), and oral frailty as the dependent variable (*Y*). Depressive symptoms is treated as the first mediating variable (M1), and nutritional risk as the second mediating variable (M2). Given that age is a confounding factor associated with oral frailty, depressive symptoms and nutritional risk, age was included as a covariate in all regression equations and mediation effect tests to control for its potential confounding effect on the associations among variables. The significance of mediating effects was assessed via Bootstrap sampling (5,000 repetitions), with 95% confidence intervals calculated at a significance level of *α* = 0.05.

## 3 Results

### 3.1 Participants characteristics

There were 500 participants who completed the questionnaires and the mean age 65.96 ± 10.40 years. The detailed results of characteristics for CHD patients are shown in Table 1.

**Table 1:** Participants characteristics (*n* = 500).

Variables	*M* ± SD/*n*(%)
Age	65.96 ± 10.40
Gender	
Male	280 (56.0)
Female	220 (44.0)
Education	
Elementary school and below	144 (28.8)
Middle school	166 (32.2)
High school or vocational school	127 (25.4)
College or higher	63 (12.6)
Monthly household income per person	
<1,000	72 (14.4)
1,000–2,999	172 (34.4)
3,000–4,999	160 (32.0)
>5,000	96 (19.2)
Residence	
Urban	272 (54.4)
Rural	228 (45.6)
Number of missing teeth	
0–1	201 (40.2)
2–4	164 (32.8)
5 and above	135 (27.0)
Polypharmacy	
Yes	398 (79.6)
No	102 (20.4)
Course of coronary heart disease	
<0.5 years	185 (37.0)
0.5–1 year	133 (26.6)
1–2 year	38 (7.6)
2 years or more	144 (28.8)

### 3.2 Scores of SSRS, PHQ-9, NRS 2002 and OF-8

Table 2 presents the descriptive statistics for the main variables. The mean score for oral frailty among CHD patients was 4.80 ± 2.86, indicating a high prevalence (57.60%) of oral frailty in this population. The mean score for social support was 37.67 ± 8.15. Additionally, the mean score for depressive symptoms was 13.41 ± 3.47, and the mean score for nutritional risk was 1.12 ± 1.13.

**Table 2:** Scores of SSRS, PHQ-9, NRS 2002 and OF-8 (*n* = 500).

Variables	Total score (*M* ± SD)	Mean item score (*M* ± SD)
SSRS	37.67 ± 8.15	3.77 ± 0.82
Objective support	9.43 ± 2.40	3.14 ± 0.80
Subjective support	21.06 ± 4.41	5.27 ± 1.10
Support utilization	7.18 ± 2.41	2.39 ± 0.80
PHQ-9	13.41 ± 3.47	1.49 ± 0.39
NRS 2002	1.12 ± 1.13	0.37 ± 0.38
Nutritional status	0.27 ± 0.52	0.27 ± 0.52
Disease severity	0.47 ± 0.73	0.47 ± 0.73
Age	0.38 ± 0.49	0.38 ± 0.49
OF-8	4.80 ± 2.86	0.60 ± 0.36
Masticatory ability	1.42 ± 1.30	0.71 ± 0.65
Swallowing function	1.39 ± 1.17	0.70 ± 0.59
Dentures use	0.38 ± 0.79	0.38 ± 0.79
Social participation	0.29 ± 0.46	0.29 ± 0.46
Oral health behaviors	1.32 ± 0.66	0.66 ± 0.33

### 3.3 Correlation analysis

We conducted correlation analysis among the variables: social support, depression, nutrition, and oral frailty in patients with coronary heart disease. The results are presented in the Table 3. The findings revealed that social support was significantly negatively correlated with depression (*r* = −0.612, *p* < 0.01), nutrition (*r* = −0.473, *p* < 0.01), and oral frailty (*r* = −0.659, *p* < 0.01). In contrast, depression was positively correlated with nutrition (*r* = 0.636, *p* < 0.01) and oral frailty (*r* = 0.603, *p* < 0.01), and nutrition was positively correlated with oral frailty (*r* = 0.600, *p* < 0.01). These results indicate that there are significant pairwise correlations among social support, depression, nutrition, and oral frailty in patients with coronary heart disease.

**Table 3:** Correlation coefficients for social support, depressive symptoms, nutritional risk and oral frailty (*n* = 500).

Variables	Social support	Objective support	Subjective support	Support utilization	Depressive symptoms	Nutritional risk	Oral frailty
Social support	1.000						
Objective support	–	1.000					
Subjective support	–	–	1.000				
Support utilization	–	–	–	1.000			
Depressive symptoms	−0.612**	−0.523**	−0.587**	−0.478**	1.000		
Nutritional risk	−0.473**	−0.455**	−0.425**	−0.373**	0.636**	1.000	
Oral frailty	−0.659**	−0.615**	−0.593**	−0.534**	0.603**	0.600**	1.000

**P < 0.01.

### 3.4 Regression analysis of the mediation model

Age was included as a covariate in the analysis to control for its potential confounding effect. After adjustment, the total effect, direct effect, and chain mediating effect of social support on oral frailty all remained statistically significant. The regression analysis indicated that social support was a direct and significant negative predictor of oral frailty [*β* = −0.132, *t*(496) = −10.647, *p* < 0.001]. Moreover, social support negatively predicted depressive symptoms [*β* = −0.229, *t*(498) = −14.533, *p* < 0.001] and nutritional risk [*β* = −0.012, *t*(497) = −1.988, *p* < 0.05]. Depressive symptoms significantly and positively predicted nutritional risk [*β* = 0.167, *t*(497) = 11.266, *p* < 0.001] and oral frailty [*β* = 0.107, *t*(496) = 3.242, *p* < 0.01]. Nutritional risk was also a significant positive predictor of oral frailty [*β* = 0.432, *t*(496) = 4.858, *p* < 0.001]. Detailed results are presented in Table 4.

**Table 4:** Regression results of the chain mediation model (*n* = 500).

Variables	Depressive symptoms		Nutritional risk		Oral frailty	
	*β*	*t*	*β*	*t*	β	*t*
Social support	−0.229	−14.533***	−0.012	−1.988*	−0.132	−10.647***
Depressive symptoms			0.167	11.266***	0.107	3.242**
Nutritional risk					0.432	4.858***
*R*	0.640		0.691		0.787	
*R^2^*	0.410		0.477		0.620	
*F*	172.357***		150.662***		201.530***	

*p < 0.05, **p < 0.01, ***p < 0.001.

**Figure 1:**
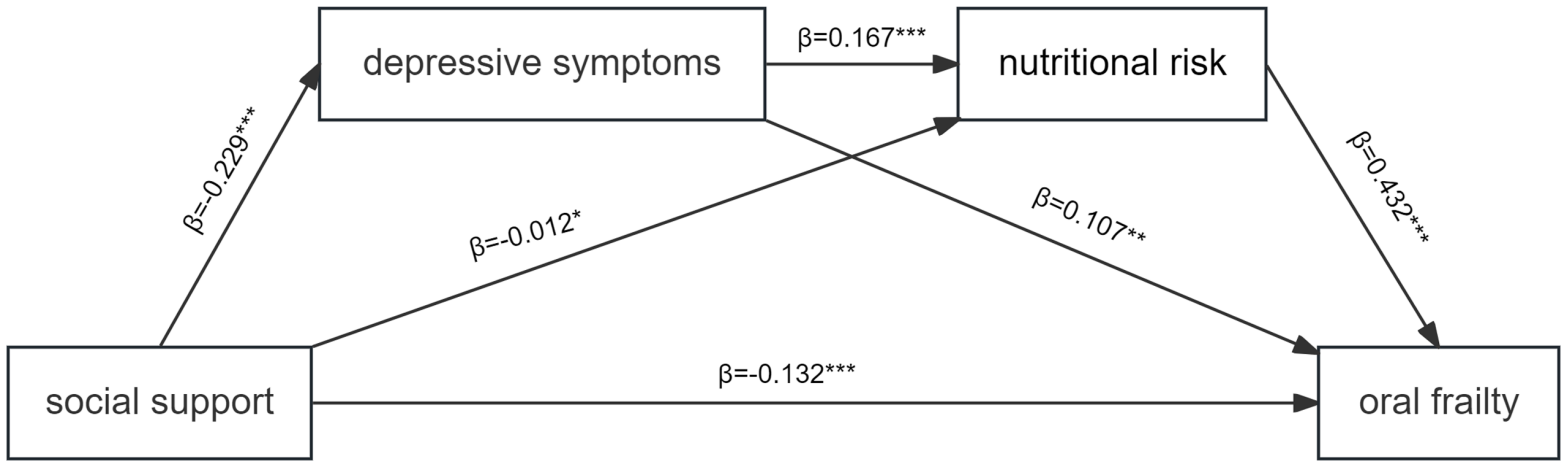
Chain mediation effect. The coefficients in figure are standardized coefficients. *p < 0.05; **p < 0.01; ***p < 0.001.

Bootstrap method revealed the mediating roles of depressive symptoms and nutritional risk in the relationship between social support and oral frailty among patients with coronary heart disease. The interaction pathways among the variables are shown in Figure 1, and the mediating effects are presented in Table 5. None of the confidence intervals included 0, confirming the validity of the partial mediating effects.

**Table 5:** Standardized direct and indirect effects (*n* = 500).

Effect type	*β*	*SE*	95%*CI*	Percentage of total effect (%)
Total effect (X → Y)	−0.178	0.011	−0.199 ~ −0.156	100%
Direct effect (X → Y)	−0.132	0.012	−0.156 ~ −0.107	74.16%
Total indirect effect	−0.046	0.008	−0.063 ~ −0.030	25.84%
Indirect path 1 (X → M1 → Y)	−0.024	0.009	−0.041 ~ −0.008	13.48%
Indirect path 2 (X → M2 → Y)	−0.005	0.003	−0.011 ~ 0.000	2.81%
Indirect path 3 (X → M1 → M2 → Y)	−0.017	0.004	−0.025 ~ −0.010	9.55%

X: social support; Y: oral frailty; M1: depressive symptoms; M2: nutritional risk.

## 4 Discussion

Consistent with the study hypotheses, the result of study identified a significant negative association between social support and oral frailty among CHD patients, and further confirmed that depressive symptoms and nutritional risk exert a significant chain mediating effect on this association. Specifically, social support can influence oral frailty through one direct pathway and three indirect pathways: the separate mediating roles of depressive symptoms and nutritional risk, and their sequential mediating pathway. These findings provide novel empirical evidence for understanding the psychosocial and physiological mechanisms underlying oral frailty, and have important implications for developing multifaceted interventions to prevent oral frailty and promote healthy aging in this vulnerable population.

The prevalence of oral frailty in this sample of hospitalized CHD patients reached 57.60%, with a mean OFI-8 score of 4.80 ± 2.86. This notable prevalence may be related to the fact that CHD patients frequently suffer from multiple chronic conditions and polypharmacy (30–32). Patients with multiple chronic diseases often have reduced physical activity and poor muscle strength, which weakens swallowing-related muscles, leading to decreased chewing function and dry mouth (33–35). The combination of multiple medications can reduce salivary secretion and exacerbate the imbalance of oral microbiota, leading to tooth loss and impaired oral function (36–38). In terms of dimensional performance, patients scored higher (indicating greater impairment) in “masticatory ability” and “swallowing function” dimensions but lower in “social participation” and “oral health behaviors,” indicating pronounced sensorimotor impairments alongside deficits in oral health maintenance awareness. In addition, the PHQ-9 score in this study reached 13.41 ± 3.47, corresponding to a moderate level of depressive symptoms. All participants were hospitalized patients, who typically faced disease exacerbation, the burden of long term polypharmacy, concerns about disease prognosis, and potential financial strain. These combined stressors substantially elevate the risk of depressive symptoms. Therefore, for CHD patients with significant depressive burden, strengthening social support to mitigate depressive symptoms may deliver a more robust protective effect on the prevention of oral frailty.

The direct effect of social support on oral frailty accounted for 74.16% of the total effect, representing the dominant pathway in this model. By acquiring emotional, instrumental, and informational support through social networks, patients can buffer the negative impacts of stressful events on their physical and mental health. Research shows that individuals who actively participate in leisure activities and maintain good family relationships exhibit higher tongue pressure, which can delay the oral frailty process (39, 40). Conversely, those with fewer conversations and chewing actions may experience a decline in oral function (41, 42). High levels of social support enhance patients’ confidence in managing complex CHD conditions, prompting them to adopt positive health behaviors and strictly adhere to oral health guidelines, thereby directly protecting oral function (43, 44). Therefore, healthcare providers should actively build social support networks for these patients to mitigate the direct risks of oral frailty.

Furthermore, social support indirectly influences oral frailty through the alleviation of depressive symptoms, accounting for 13.48% of the total effect. CHD patients frequently face challenges such as disease uncertainty and strict lifestyle restrictions, which easily trigger depressive symptoms. Depression can lead to self-neglect, reducing patients’ attention to oral hygiene, thereby increasing the risk of dental plaque accumulation and oral diseases, ultimately exacerbating oral frailty (17). Depressive symptoms also lower self-efficacy, causing a regression in health behaviors. Adequate social support can reduce loneliness, stabilize mood, and prevent depression from interfering with oral health self-management (45), acting as an indirect protective mechanism.

Another pathway is that nutritional risk acts as a mediator between social support and oral frailty, accounting for 2.81% of the total effect. CHD patients must follow cardiovascular health dietary guidelines while maintaining sufficient nutritional intake, and social support can effectively reduce the burden of nutritional management and lower nutritional risk (46). Nutritional risk exerts a stronger effect on oral frailty than any other factor in the model. While social support and depressive symptoms represent psychosocial and behavioral determinants, nutritional status is the core physiological factor underlying oral frailty. Malnutrition directly impairs masticatory muscle strength and the repair capacity of the oral mucosa by reducing systemic muscle mass (47); it also alters salivary composition and elevates the risk of oral infections (48). The impact of this direct biological association is far more pronounced than that of psychosocial factors. This suggests that this cycle can be disrupted by ensuring adequate intake of essential nutrients through social support, thereby consolidating the physiological foundation of oral health.

The study findings suggest that social support delays the progression of oral frailty in CHD patients through a chain mediating pathway where reduced depressive symptoms lead to lowered nutritional risk, accounting for 9.55% of the total effect. This pathway aligns with the Gobbens’ integrative theory of frailty (11). Insufficient social support acts as the initial factor, affecting the behavioral mediator of nutritional intake through the psychological mediator of depressive symptoms, ultimately impacting the physical outcome of oral frailty. Therefore, in clinical practice, comprehensive interventions are paramount. Healthcare providers should not only focus on localized oral health but also heavily weigh psychosocial factors and nutritional status. The chain mediating pathway can be interrupted by enhancing social support, promptly screening for depressive symptoms, and addressing nutritional risk.

## 5 Limitation

This study has several limitations. First, this was a single center study, and all participants were recruited from hospitalized patients with CHD, which may limit the generalizability of the results. Future investigations should adopt multi-center collaboration to increase diversity and validate findings. Second, as a cross-sectional study, it cannot confirm a causal relationship between social support and oral frailty. Consequently, longitudinal studies are needed to further explore the strength of associations between variables. Lastly, the mean PHQ-9 score in the sample fell within the moderate depression range, which may limit the generalizability of findings to who with lower depressive symptoms. Accordingly, replication in samples with diverse depression levels is warranted.

## 6 Conclusion

This study systematically explores the pathways between social support and oral frailty in CHD patients, and helps elucidate the complex interactions among psychosocial factors, nutritional risk, and oral health in this population. The results demonstrate that social support may functions as a protective factor against oral frailty through two pathways: a direct protective effect and an indirect sequential mediating effect via depressive symptoms and nutritional risk. These findings provide preliminary evidence to inform the development of multifaceted interventions for preventing oral frailty in CHD patients. Based on the identified pathways, healthcare providers may implement routine screening for social support, depressive symptoms, nutritional risk and oral frailty as part of standard cardiology care to facilitate early identification of risk patients. Multidisciplinary teams comprising cardiologists, dentists, psychologists and dietitians could collaborate to deliver integrated interventions targeting the identified mediating pathway.

## Data Availability

The raw data supporting the conclusions of this article will be made available by the authors, without undue reservation.
